# RETRACTED: Tang et al. The Hidden Danger of Unintentional Child Injuries in an Urban Domestic Environment: Considering Unintentional Injuries from Another Angle. *Int. J. Environ. Res. Public Health* 2025, *22*, 1068

**DOI:** 10.3390/ijerph23060770

**Published:** 2026-06-08

**Authors:** Ping Tang, Qin Fan, Jingmin Sun, Jianlin Ji, Liling Yang, Wenjuan Tang, Qunfeng Lu

**Affiliations:** 1 Nursing Department of Children’s Hospital, School of Medicine, Shanghai Jiao Tong University, Shanghai Children’s Hospital, Shanghai 200062, China; tangping@shchildren.com.cn; 2 General Surgery Department of Children’s Hospital, School of Medicine, Shanghai Jiao Tong University, Shanghai Children’s Hospital, Shanghai 200062, China; fanq@shchildren.com.cn; 3 Outpatient Department/Emergency Department of Children’s Hospital, School of Medicine, Shanghai Jiao Tong University, Shanghai Children’s Hospital, Shanghai 200062, China; sunjm@shchildren.com.cn; 4 School of Nursing, School of Medicine, Shanghai Jiao Tong University, Shanghai 200025, China; jijianlin@sjtu.edu.cn; 5 Nursing Department of the Sixth People’s Hospital, School of Medicine, Shanghai Jiao Tong University, Shanghai 200233, China; youngll955@163.com

The journal retracts the article titled “The Hidden Danger of Unintentional Child Injuries in an Urban Domestic Environment: Considering Unintentional Injuries from Another Angle” [1], cited above.

Following publication, concerns were brought to the attention of the Editorial Office regarding the accuracy of ethical approval documentation and appropriate ethical oversight of the study reported in this publication [1].

In accordance with the journal’s standard procedures, the Editorial Office and Editorial Board conducted an investigation, which identified inconsistencies in the reported sample recruitment and size as well as the overall scope of the study between the published article and the ethics documentation provided by the authors. As a result of these inconsistencies, the Editorial Board were unable to determine with confidence whether the published study was appropriately covered by the ethical approval documentation submitted. Given the importance of clear and verifiable ethical oversight for research involving human participants, the Editors no longer have confidence in the ethical oversight of the study and have therefore decided to retract the article as per the MDPI retraction policy.

This retraction was approved by the Editor-in-Chief of the journal *IJERPH*. 

The authors have been informed of this retraction and have indicated their agreement with this decision.
